# Road to Hierarchical Diabetes Management at Primary Care (ROADMAP) Study in China: Protocol for the Statistical Analysis of a Cluster Randomized Controlled Trial

**DOI:** 10.2196/18333

**Published:** 2020-04-28

**Authors:** Xian Li, Nadila Duolikun, Fengzhuo Cheng, Laurent Billot, Weiping Jia, Puhong Zhang

**Affiliations:** 1 The George Institute for Global Health at Peking University Health Science Center Beijing China; 2 Faculty of Medicine University of New South Wales Sydney Australia; 3 The George Institute for Global Health Sydney Australia; 4 Department of Endocrinology Shanghai Jiaotong University Affiliated Sixth People’s Hospital Shanghai China; 5 Chinese Diabetes Society Beijing China

**Keywords:** statistical analysis plan, cluster randomized controlled trial, diabetes control, China, community-based, hemoglobin, primary care

## Abstract

**Background:**

As the management of type 2 diabetes remains suboptimal in primary care, the Road to Hierarchical Diabetes Management at Primary Care (ROADMAP) study was designed and conducted in diverse primary care settings to test the effectiveness of a three-tiered diabetes management model of care in China.

**Objective:**

This paper aims to predetermine the detailed analytical methods for the ROADMAP study before the database lock to reduce potential bias and facilitate transparent analyses.

**Methods:**

The ROADMAP study adopts a community-based, cluster randomized controlled trial design that compares the effectiveness of a tiered diabetes management model on diabetes control with usual care among patients with diabetes over a 1-year study period. The primary outcome is the control rate of glycated hemoglobin (HbA_1c_) <7% at 1 year. Secondary outcomes include the control rates of ABC (HbA_1c_, blood pressure, and low-density lipoprotein cholesterol [LDL-C], individual and combined) and fasting blood glucose, and the change in each outcome. The primary analysis will be the log-binomial regression with generalized estimating equation (GEE), which accounts for the clustering within communities, for binary outcomes and linear regression with GEE for continuous outcomes. For both, the baseline value of the analyzed outcome will be the covariate. The other covariate further adjusted models and the repetitive models after multiple imputation (when more than 10% of observations in HbA_1c_ after 1 year are missing) will be used for sensitivity analysis. Five prespecified subgroup analyses have also been planned to explore the heterogeneity of the intervention effects by adding the subgroup variable and its interaction with the intervention to the primary model.

**Results:**

This plan has been finalized, approved, and signed off by the principle investigator, co-principle investigator, and lead statisticians as of November 22, 2019, and made public on the institutional website without any knowledge of intervention allocation. Templates for the main figure and tables are presented.

**Conclusions:**

This statistical analysis protocol was developed for the main results of the ROADMAP study by authors blinded to group allocation and with no access to study data, which will guarantee the transparency and reduce potential bias during statistical analysis.

**Trial Registration:**

Chinese Clinical Trial Registry ChiCTR-IOC-17011325; https://tinyurl.com/ybpr9xrq

**International Registered Report Identifier (IRRID):**

DERR1-10.2196/18333

## Introduction

### Study Background

Being home to the largest diabetic population, China has been encountering challenges in managing diabetes adequately in primary care [1-3]. Currently, patients with type 2 diabetes (T2D) are entitled to four or more yearly free blood glucose tests and treatment consultations at primary care clinics through a publicly funded essential public health service package [4]. Despite the provision of the universal access and increasing subsidies to community health care services [4,5], outcomes of the current management remain suboptimal. It has been reported that among service recipients only 40% have reached the adequate blood glucose control target (glycated hemoglobin [HbA_1c_] <7.0%) [6], while less than a tenth have achieved optimal control of composite cardiometabolic “ABC” (HbA_1c_, blood pressure [BP], and low-density lipoprotein cholesterol [LDL-C]) targets [7]. In July 2017, a cluster randomized controlled trial, Road to Hierarchical Diabetes Management at Primary Care (ROADMAP) study, was launched to determine the effectiveness of a strengthened version of the previously mentioned essential public health service on diabetes management through a mobile health–based, tiered service-delivery intervention in diverse primary care settings in China.

### Study Overview

The ROADMAP study is designed as a community-based, cluster randomized controlled trial, aiming to compare the effectiveness of a hierarchical diabetes management intervention to usual care on blood glucose control. The usual care is the routine diabetes and hypertension management required by the national essential public health service. [4] The intervention delivery is performed by contracted service teams; each of them are composed of one primary care doctor at the community or village level as team leader, one township hospital doctor, and one district or county doctor. The intervention lasted for 1 year. This study is prospectively registered (ChiCTR-IOC-17011325) and ethically approved. A complete study description has been published elsewhere [8].

### Participant Recruitment and Randomization

Participants are adult patients with established T2D who have registered for the essential public health service within the community at the time of recruitment. To be eligible, participants were 18-75 years old, resided in the community for the previous 6 months with no plan of relocating, and provided informed consent. Potential participants were excluded if they had severe physical or psychological injury or illness, were unable to attend the site visit or consciously answer questions, were women in the process of or planning for pregnancy or breastfeeding, or had participated in any other clinical trial within the previous 6 months.

The trial recruited a total of 19,601 participants from 864 communities or villages in 144 districts or counties in 25 provinces. Generally, for each participating province, 6 districts and 6 of its subordinate communities from each district were selected. The same principle applied to counties and villages in rural areas. Following the completion of baseline assessments, communities or villages (clusters) were centrally randomized.

### Intervention

Besides a standard training workshop for the contracted service providers (community, township, and county level doctors) in the intervention arm, the key components of intervention were one BP measurement and two blood glucose monitoring tests (at least one fasting blood glucose [FBG]) monthly, instruction for lifestyle change and medication accordingly, timely referral if an indicator is present, and quarterly performance review for the contracted service team. A mobile health–based information system, Graded ROADMAP, was developed and employed to support the contracted doctor team delivering the intervention. Another smartphone app, Your Doctor, was available for participants in the intervention arm to facilitate health education and communication between the designated doctors and patients. The use of Your Doctor depended on participants’ willingness and capability of using the smartphone app. At the end of this study, all the participants in the intervention arm were divided into two subgroups based on the actual use of Your Doctor: a basic intervention subgroup in which participants have logged in less than 4 times to the app throughout the 1-year follow-up and an intensive intervention subgroup in which the participants have at least 4 log-ins within 1 year.

### Outcomes

The primary outcome is the HbA_1c_ control rate (percentage of patients achieving HbA_1c_ <7.0%; target A) at 1 year. The secondary outcomes include the percentage of patients achieving both systolic blood pressure <140 mmHg and diastolic blood pressure <80 mmHg (target B); the percentage of patients achieving LDL-C <2.6 mmol/L (target C); the optimal control rate of the combined ABC targets as previously defined; the percentage of patients achieving FBG <7.0 mmol/L; the changes in levels of HbA_1c_, BP, LDL-C, and FBG; and the subtype-specific and overall hypoglycemia episodes [9]. Other outcomes are health-related quality of life measured by the EuroQol questionnaire EQ-5D-3L (3-level version of EQ-5D) [10,11], the mean change in the scores of the summary of diabetes self-care activities questionnaire [12], the development of any self-reported onset of new comorbidities and diabetic complications during follow-up, concomitant medications, and direct medical cost.

### Sample Size

A sample size of 16,416 participants (19 patients per community) at 1 year provided an 89% power (2-sided α=.05) to detect a ≥5% absolute increase in the primary outcome for the intervention group. The sample size calculation assumes that 40% of participants will have well-controlled HbA_1c_ levels (<7%) at the end of the study in the control group [6], with an intraclass correlation coefficient of 0.15 based on our previous Observational Registry of Basal Insulin Treatment (ORBIT) study [13]. Furthermore, assuming that 50% of participants (ie, 576 clusters with a smaller average cluster size of 9-10 participants) in the intervention group will receive the intensive intervention (at the patients’ discretion), it will need 93% to detect absolute increases of 5% HbA_1c_ control, when compared to the basic intervention group (576 clusters with an average cluster size of 9-10 patients). Accounting for a potential loss to follow-up of 14% of patients, the study aimed to recruit 19,008 patients with T2D from 864 communities or villages (576 in intervention and 288 in control, with a 2:1 ratio) in 24 provinces in mainland China, which equates to an average of 22 patients from each community or village.

### Objectives

The purpose of this study is to outline the predetermined analytical methods in detail before completion of the database lock to reduce the potential bias and facilitate transparent analyses.

## Methods

### Patient Disposition

**Figure 1:**
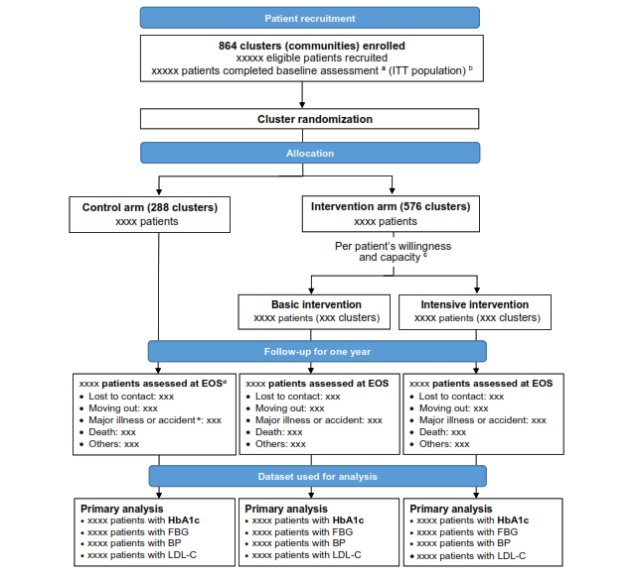
CONSORT diagram for the randomized control trial. BP: blood pressure; EOS: end of study; FBG: fasting blood glucose; HbA_1c_: glycated hemoglobin; ITT: intention-to-treat; LDL-C: low-density lipoprotein cholesterol.

The flow of patients through the study will be displayed in a CONSORT (Consolidated Standards of Reporting Trials) diagram (figure shell is shown in Figure 1).

### Population for Analysis

The main analysis will be performed at the patient level following an intention-to-treat (ITT) principle (ie, patients and clusters will be included for analysis as their assigned group, regardless of treatment adherence). All randomized patients that have given consent and are not missing key variables (including gender, age, and HbA_1c_ level) at the baseline assessment will form the ITT population.

### General Analysis Principles

Analyses will adjust for clustering at the village level. The intracluster correlation coefficients will be calculated and tabulated. The primary comparison will be made between the intervention population and the usual care population. As a secondary comparison, the effects of the intensive intervention (no less than 4 logins to the Your Doctor app in addition to the basic intervention) will be explored by estimating the effects of intensive intervention vs basic intervention. No formal interim analysis will be performed. The primary analysis will use all available data with no imputation. Imputation will be performed as a sensitivity analysis when missing observations in HbA_1c_ levels after 1 year exceed 10%.

Hypothesis tests will be 2-tailed with a 5% significance level maintained throughout the analyses. No adjustment will be applied for multiplicity, given that most of the effectiveness outcomes are correlated or consist in different versions of common variables. Outcomes will be presented in order of their priority (primary vs secondary), and only a limited number of subgroup analyses are prespecified. Subgroup analyses will be carried out irrespective of whether a significant treatment effect on the primary outcome is observed. Analyses will be performed in SAS, version 9.4 or later (SAS Institute).

### Patient Characteristics and Baseline Comparison

Discrete baseline variables will be summarized by frequencies and percentages. Percentages will be calculated using the number of patients for whom data is available. Continuous variables will be summarized by using mean and SD, and median and interquartile range (Q1-Q3). No adjustment for clustering will be applied when comparing baseline characteristics. Standardized differences between groups in baseline characteristics will be reported. A less than 0.1 standard difference will be used to indicate a negligible difference in the mean or proportion of a baseline variable between treatment groups [14].

Variables for baseline measures will be tabulated, including demographic (age, sex, ethnicity), socioeconomic status (education level, household income, health insurance), anthropometric measurements (weight, BMI, waist circumference, BP), smoking status, diabetic complications, laboratory data (HbA_1c_, FBG, LDL-C, serum creatinine), and score of the summary of diabetes self-care activities.

Cluster characteristics will be summarized in stratified and overall randomization groups. The cluster characteristics at baseline are age of the community or village doctors, smartphone operation system (iOS or Android) that the community or village doctors were using, and number of patients with T2D registered.

### Compliance to Basic Intervention Service

Data on the receipt of intervention provided for standardized diabetes management services have been routinely collected by the app Graded ROADMAP, including the frequencies and the results of FBG or postprandial blood glucose tests, BP measurements, and patient referrals from primary care clinics to the upstream hospitals at the county or district level. According to the protocol, each patient is due to receive at least two blood glucose tests (FBG, postprandial blood glucose, or both) and one BP measurement per month. Although necessary referral is encouraged when indicators are present, there is no requirement for the referral rate.

Among the intervention group, the mean times of health services received per patient per month; the percentage of patients achieving the protocol-required measurement frequency; and the mean values of FBG, postprandial blood glucose, and BP will be illustrated in separate figures with a double y-axis.

### Exposure to Intensive Intervention

Within the intervention group, the frequency of patient’s log-ins to the Your Doctor app will be described in numbers and percentages of frequency from 0 to >12 over the 1-year study period, and as a categorical variable in frequency ≥4 or <4. A patient with the frequency ≥4 times per year (referring to the essential public health service recommendation) will be defined as a complier to intensive intervention and, thus, form the intensive intervention subset for further comparison between basic and intensive intervention.

### Analysis of the Primary Outcome

#### Primary Analyses

The primary endpoint, control rate of HbA_1c_ at 1 year, will be first compared between all intervention groups and all control groups. The primary analysis of the intervention effect will be conducted using a log-binomial regression with generalized estimating equations (GEE) to account for clustering within communities and with adjustment for baseline HbA_1c_ as a continuous covariate (model 1). The raw number and percentage of patients with adequate control of HbA_1c_ at 1 year will be reported. The effect of the intervention will be presented as the relative risk (RR) of the proportion of HbA_1c_ levels <7% along with its 95% CI and corresponding *P* value. In cases of a convergence issue, the logistic regression with GEE and the Poisson regression with GEE will be both used as the alternative methods, with the Poisson regression as the sensitivity analysis. The odds ratio along with the indirectly derived RR will be reported for the logistic regression.

#### Covariates-Adjusted Analyses

The primary model (model 1) previously described will be rerun after further adjusting for the following covariates (model 2): economic development level; locality; and other baseline covariates that have shown a significant difference (*P*<.01) between the intervention and control groups in a univariate comparison, including all the baseline variables previously listed (see “Patient Characteristics and Baseline Comparison”).

#### Imputed Data Analyses

Multiple imputation using fully conditional specification [15] will be performed as a sensitivity analysis when more than 10% of observations in HbA_1c_ is missing at 1 year. The imputation model will include the levels of HbA_1c_, FBG, BP, and LDL-C at 1 year and at baseline; a cluster indicator; a group indicator; and other baseline variables including age, sex, education, duration of diabetes, comorbidities, economic development level, and locality (urban or rural). Ten sets of imputed data will be created and analyzed using the model 1 (described in “Primary Analyses”). HbA_1c_, FBG, BP, and LDL-C will first be imputed as continuous variables using linear regression and subsequently converted into binary variables. Estimates of the intervention effect after imputation (β in model 1) and its standard errors will be combined to obtain a pooled common RR and 95% CI.

#### Subgroup Analyses

Five prespecified subgroup analyses will be carried out between the overall intervention group and the control group. The subgroups are economic development level (developed vs less-developed), locality (urban vs rural), age group (<60 years of age vs ≥60 years of age), duration of diabetes (≥6 years vs <6 years, around the median), and diabetic complication (yes vs no; yes is defined as presence of any diagnosed diabetic nephropathy, diabetic retinopathy, peripheral neuropathy, carotid artery disease, lower extremity artery disease, diabetic foot damage, peripheral vascular disease, coronary stenosis, myocardial infarction, postcoronary artery surgery, cerebral infarction, or cerebral hemorrhage).

The analysis for each subgroup analysis will be performed by adding the subgroup variable along with its interaction with the intervention as fixed effects to the primary model (model 1). Within each subgroup, the raw counts and percentages within each treatment arm will be presented, as well as the RRs and their 95% CIs for the intervention effect from the primary model. The results will be displayed on a forest plot including the *P* value for heterogeneity corresponding to the interaction term between the intervention and the subgroup variable.

#### Comparison Between Basic and Intensive Intervention

To explore the possible additional effect from intensive intervention, we will compare the intensive intervention to the basic intervention using similar models, regardless of whether the difference between the overall intervention group and control group is statistically significant. Considering the potential imbalance in the baseline characteristics between patients following the intensive intervention and those following the basic intervention, two propensity score methods will be applied. Both the propensity score adjusted regression based on a log-binomial model with GEE and the inverse probability of treatment weighting method will be used to evaluate the intervention effect [16,17] as the sensitivity to seek the consistency of the conclusion. The propensity score model will consist in a simple logistic regression with baseline covariates including age, sex, the baseline value of the analyzed outcome, education, duration of diabetes, comorbidities, economic development level, and locality (urban or rural). Baseline characteristics of participants will be described in a table separately before and after propensity score adjustments.

### Analysis of Secondary Outcomes

#### Analysis for Binary Categorical Outcomes

A similar analytic strategy as for the primary outcome will be followed for other binary outcomes. These include the proportion of FBG<7.0 mmol/L, BP<140/80 mmHg, and that of LDL-C<2.6 mmol/L, and the optimal control rate of combined ABC targets. A log-binomial regression with GEE and including baseline continuous values of the analyzed outcome variable as covariate (model 1) will be used. Other further adjusted analysis (model 2) and subgroup analysis will also be conducted, and the imputed analysis as well, if applicable.

#### Analysis for Continuous Outcomes

HbA_1c_, FBG, BP, and LDL-C will also be analyzed as continuous variables. A similar analytic strategy as the one used for binary outcomes will be followed but using a linear regression (ie, assuming a normal distribution and an identity link, instead of a log-binomial regression). The raw mean (SD) of the changes will be reported. The effect of the intervention will be presented as the adjusted mean difference and associated 95% CIs. Further covariates-adjusted analyses (see model 2) and subgroup analysis will also be conducted, and the imputed analysis as well, if applicable.

#### Analysis of Hypoglycemia

Episodes of hypoglycemia (each subtype and overall) will be analyzed with the same approach as before; this time using Poisson regression adjusted for the baseline count of hypoglycemia. The effect of the intervention will be estimated as the incidence rate of hypoglycemia episodes and its 95% CI. The number of patients experiencing at least one hypoglycemia episode and the total number of episodes will be tabulated by group. Adjusted analysis and subgroup analysis will also be conducted. No imputation will be performed on hypoglycemia.

### Analysis of Other Outcomes

No subgroup and imputed analysis will be performed on the following endpoints.

#### EQ-5D

The EQ-5D index value and EuroQol-visual analogue scale (EQ-VAS) score at baseline and at end-of-study will be described using mean and SD by treatment groups. For changes in EQ-5D index values and VAS scores, a linear regression with GEE accounting for clustering will be used to test the difference between groups. The baseline values of the outcome variable will be included as covariates. The raw mean (SD) of the score changes will be reported by treatment groups; the effect of the intervention will be presented as the mean differences of the changes and associated 95% CIs.

#### The Summary of Diabetes Self-Care Activities

The scores of the summary of diabetes self-care activities questionnaire will be described at baseline and 1 year. For their changes from baseline, a linear regression with GEE and with adjustment of the baseline values of analyzed outcome variables will be used to compare the difference between treatment groups. Other covariates with further adjusted models will also be conducted. The raw mean (SD) of the score changes will be reported by treatment groups; the effect of the intervention will be presented as the mean differences and associated 95% CIs from the two previously mentioned models.

#### Other Variables

The following outcomes will be analyzed using descriptive statistics without any adjustment for clustering.

1. Concomitant medications. Insulin injection and oral antidiabetic drug intake at baseline and at end-of-study assessments. The combination of different oral antidiabetic drug regimens or the combination of insulin injection and oral antidiabetic drugs, or their single use will also be summarized.
2. New-onset comorbidities and diabetic complications will be described as the numbers and percentages of patients with each new onset complication by treatment groups.
3. Direct medical cost is the self-reported direct cost on medication and medical expense for health care services (inpatient or outpatient cost, medication cost), including total cost and out-of-pocket cost.

## Results

This study was funded in January 2017. Ethics approval was obtained from the Institutional Review Board at Shanghai Sixth People's Hospital, where the lead PI is affiliated with, before the study commenced. Written approval from each participating site was granted by the local hospital research ethics committee, and other relevant regional regulatory bodies. Signed informed consent was obtained from all trial participating doctors and patients prior to participant recruitment. Recruitment commenced in June 2017 and closed after completed baseline assessment in December 2018 for all 864 trial participating communities in 144 districts or counties in 25 provincial sites (1 more province than the scheduled 24 because of a shortage in eligible district or county hospitals). As of October 2019, the last 1-year end-of-study assessment ended. The internal statistical plan was reviewed, approved, and signed off in November 2019 and made public on the institutional internal website prior to the database lock in January 2020.

Templates of main tables (ie, baseline characteristics as in Table 1, estimated intervention effects for binary outcomes as in Table 2, estimated intervention effects for continuous outcomes as in Table 3, and hypoglycemia incidence as in Table 4) were produced prior to the previously mentioned analytical methods.

**Table 1:** Baseline characteristics of participants by treatment arms in the road to hierarchical diabetes management at primary care study.

Characteristics				Control (xxxx)		Intervention (xxxx)		Standardized differences
**Region by economic development, n (%)**							x.xxx	
	Developed		xxx (xx.x)		xxx (xx.x)			
	Less developed		xxx (xx.x)		xxx (xx.x)			
**Locality, n (%)**							x.xxx	
	Urban		xxx (xx.x)		xxx (xx.x)			
	Rural		xxx (xx.x)		xxx (xx.x)			
**Demographics**								
	Age (years), mean (SD)		xx (xx)		xx (xx)		x.xxx	
	Gender (male), n (%)		xxx (xx.x)		xxx (xx.x)		x.xxx	
	BMI (kg/m^2^), mean (SD)		xxx (xxx)		xxx (xxx)		x.xxx	
	**Highest level of education, n (%)**						x.xxx	
		Primary school or lower	xxx (xx.x)		xxx (xx.x)			
		Junior high school	xxx (xx.x)		xxx (xx.x)			
		Senior high school	xxx (xx.x)		xxx (xx.x)			
		Junior college and above	xxx (xx.x)		xxx (xx.x)			
	Annual income per capita (CNY), mean (SD)		xxx (xxx)		xxx (xxx)		x.xxx	
	Health insurance coverage (Yes), n (%)		xxx (xx.x)		xxx (xx.x)		x.xxx	
	**Insurance reimbursement rates, n (%)**						x.xxx	
		70%-100%	xxx (xx.x)		xxx (xx.x)			
		50%-70%	xxx (xx.x)		xxx (xx.x)			
		<50%	xxx (xx.x)		xxx (xx.x)			
**Self-reported medical history and complications**								
	Duration of diabetes (years), median (Q1, Q3)		xxx (xxx, xxx)		xxx (xxx, xxx)		x.xxx	
	Current smoker, n (%)		xxx (xx.x)		xxx (xx.x)		x.xxx	
	Hypertension, n (%)		xxx (xx.x)		xxx (xx.x)		x.xxx	
	Dyslipidemia, n (%)		xxx (xx.x)		xxx (xx.x)		x.xxx	
	Diabetic nephropathy, n (%)		xxx (xx.x)		xxx (xx.x)		x.xxx	
	Diabetic retinopathy, n (%)		xxx (xx.x)		xxx (xx.x)		x.xxx	
	Peripheral neuropathy, n (%)		xxx (xx.x)		xxx (xx.x)		x.xxx	
	Lower extremity, n (%)		xxx (xx.x)		xxx (xx.x)		x.xxx	
	Macro-vascular, n (%)		xxx (xx.x)		xxx (xx.x)		x.xxx	
**Lab characteristics**								
	HbA_1c_^a^ (%), mean (SD)		x.xx (x.xx)		x.xx (x.xx)		x.xxx	
	HbA_1c_<7%, n (%)		xxx (xx.x)		xxx (xx.x)		x.xxx	
	FBG^b^ (mmol/L), mean (SD)		x.xx (x.xx)		x.xx (x.xx)		x.xxx	
	FBG<7.0 mmol/L, n (%)		xxx (xx.x)		xxx (xx.x)		x.xxx	
	SBP^c^ (mmHg), mean (SD)		xxx (x.xx)		xxx (x.xx)		x.xxx	
	DBP^d^ (mmHg), mean (SD)		xxx (x.xx)		xxx (x.xx)		x.xxx	
	LDL-C^e^ (mmol/L), mean (SD)		x.xx (xxx)		x.xx (xxx)		x.xxx	
	LDL-C<2.6 mmol/L, n (%)		xxx (xx.x)		xxx (xx.x)		x.xxx	
	Serum creatinine (umol/L), median (Q1, Q3)		xxx (xxx, xxx)		xxx (xxx, xxx)		x.xxx	

^a^HbA_1c_: glycated hemoglobin.

^b^FBG: fasting blood glucose.

^c^SBP: systolic blood pressure.

^d^DBP: diastolic blood pressure.

^e^LDL-C: low-density lipoprotein cholesterol.

**Table 2:** Estimated effects of intervention compared to control on primary and secondary binary outcomes at end of study.

Outcomes			Control, n (%)	Intervention, n (%)	Primary model^a^	
					RR^b^ (95% CI)	*P* value
**Primary outcome**						
	HbA_1c_^c^ < 7.0%		xxx (xx.x)	xxx (xx.x)	x.xx (x.xx-x.xx)	.xx
**Secondary outcomes**						
	FBG^d^ < 7.0 mmol/L		xxx (xx.x)	xxx (xx.x)	x.xx (x.xx-x.xx)	.xx
	BP^e^ < 140/80 mmHg^f^		xxx (xx.x)	xxx (xx.x)	x.xx (x.xx-x.xx)	.xx
	LDL-C^g^ < 2.6 mmol/L		xxx (xx.x)	xxx (xx.x)	x.xx (x.xx-x.xx)	.xx
	Composite diabetes control^h,i^		xxx (xx.x)	xxx (xx.x)	x.xx (x.xx-x.xx)	.xx

^a^Primary model: log-binomial regression with generalized estimating equation (GEE) with adjustment of the baseline value of the analyzed outcome and clustering. The logistic regression with GEE will be employed as the alternative method in case of non-convergence, with indirectly derived relative risk reported.

^b^RR: relative risk.

^c^HbA_1c_: glycated hemoglobin.

^d^FBG: fasting blood glucose.

^e^BP: blood pressure.

^f^Only systolic blood pressure at baseline and clustering were adjusted in the primary model for BP control.

^g^LDL-C: low-density lipoprotein cholesterol.

^h^Composite diabetes control: defined as HbA_1c_ level <7.0%, BP <140/80 mmHg and LDL-C <2.6 mmol/L.

^i^No baseline variable was adjusted in the primary model for the composite diabetes control.

**Table 3:** Estimated effects of intervention compared to control on the change from baseline of continuous outcomes.

Secondary continuous outcome		Control, mean (SD)	Intervention, mean (SD)	Primary model^a^	
				Mean differences (95% CI)	*P* value
**The change from baseline of:**					
	HbA_1c_^b^ level, %	x.xx (x.xx)	x.xx (x.xx)	x.xx (x.xx-x.xx)	.xx
	FBG^c^ level, mmol/L	x.xx (x.xx)	x.xx (x.xx)	x.xx (x.xx-x.xx)	.xx
	Systolic blood pressure, mmHg	x.xx (x.xx)	x.xx (x.xx)	x.xx (x.xx-x.xx)	.xx
	Diastolic blood pressure, mmHg	x.xx (x.xx)	x.xx (x.xx)	x.xx (x.xx-x.xx)	.xx
	LDL-C^d^ level, mmol/L	x.xx (x.xx)	x.xx (x.xx)	x.xx (x.xx-x.xx)	.xx

^a^Primary model: linear regression with generalized estimating equation and with adjustment of baseline value of the analyzed outcome and clustering.

^b^HbA_1c_: glycated hemoglobin.

^c^FBG: fasting blood glucose.

^d^LDL-C: low-density lipoprotein cholesterol.

**Table 4:** Incidence and events rate of hypoglycemia by treatment arms.

Hypoglycemia^a^	Control (n=xxxx)			Intervention (n=xxxx)				*P* value^b^	
	Patients	Events	Events/100 patients		Patients	Events	Events/100 patients		
Symptomatic hypoglycemia	xxxx	xxxx	xx		xxxx	xxxx	xx		.xx
Asymptomatic hypoglycemia	xxxx	xxxx	xx		xxxx	xxxx	xx		.xx
Probable symptomatic hypoglycemia	xxxx	xxxx	xx		xxxx	xxxx	xx		.xx
Relative hypoglycemia	xxxx	xxxx	xx		xxxx	xxxx	xx		.xx
Overall hypoglycemia	xxxx	xxxx	xx		xxxx	xxxx	xx		.xx

^a^Hypoglycemia subtypes followed the American Diabetes Association and The Endocrine Society suggested classifications [9].

^b^*P* values will be reported from Poisson regression with generalized estimating equation and with adjustment of baseline count of each hypoglycemia category.

## Discussion

### Summaries

This article presents the detailed statistical analysis plan for the ROADMAP study, which is a clustered randomized controlled trial conducted in diverse areas of China with the purpose of testing the effectiveness of a mobile health platform named *Graded Roadmap* on diabetes control.

The clustered randomized controlled trial design is useful for assessing community-based interventions like the ROADMAP study yet requires careful attention to conduct valid analyses. As such, clustering of outcomes was accounted for when designing the study and, as previously mentioned, will be accounted for in the analysis of the study outcomes. In the study design, the intraclass correlation coefficient was estimated to be 0.15 based on our previous ORBIT study. ORBIT recruited patients who were initiating basal insulin treatment at secondary and tertiary hospitals. The intraclass correlation coefficient may differ from that observed in the studied populations in ROADMAP.

To find out the effectiveness and feasibility of the platform in different regions, four strata were covered by ROADMAP. They are economically developed urban areas, economically developed rural areas, economically less-developed urban areas, and economically less-developed rural areas. Although the economic development level (developed vs less-developed) and locality (urban vs rural) have been included in the predetermined subgroup analysis, more detailed descriptions might be needed for the factorial subgroups.

### Conclusions

This statistical analysis plan was developed for the main results of the ROADMAP study by authors blinded to group allocation and with no access to study data, which will guarantee the transparency and reduce potential bias during statistical analysis.

## Appendix

Multimedia Appendix 1 CONSORT-eHEALTH checklist (V 1.6.1).

## Abbreviations

ABC: HbA_1c_, BP, and LDL-C
BP: blood pressure
CONSORT: Consolidated Standards of Reporting Trials
DBP: diastolic blood pressure
EQ-5D-3L: 3-level version of EQ-5D
EQ-VAS: EuroQol-visual analogue scale
FBG: fasting blood glucose
GEE: generalized estimating equation
HbA_1c_: glycated hemoglobin
ITT: intention-to-treat
LDL-C: low-density lipoprotein cholesterol
ORBIT: Observational Registry of Basal Insulin Treatment
ROADMAP: Road to Hierarchical Diabetes Management at Primary Care
RR: relative risk
T2D: type 2 diabetes

## References

[1] (2019). International Diabetes Federation.

[2] Zhou M, Wang H, Zeng X, Yin P, Zhu J, Chen W, Li X, Wang L, Wang L, Liu Y, Liu J, Zhang M, Qi J, Yu S, Afshin A, Gakidou E, Glenn S, Krish VS, Miller-Petrie MK, Mountjoy-Venning WC, Mullany EC, Redford SB, Liu H, Naghavi M, Hay SI, Wang L, Murray CJL, Liang X (2019). Mortality, morbidity, and risk factors in China and its provinces, 1990-2017: a systematic analysis for the Global Burden of Disease Study 2017. Lancet.

[3] Mao W, Yip CW, Chen W (2019). Complications of diabetes in China: health system and economic implications. BMC Public Health.

[4] (2017). National Health and Family Planning Commission, P.R.China.

[5] (2019). Ministry of Finance, P.R. China.

[6] Xu Y, Wang L, He J, Bi Y, Li M, Wang T, Wang L, Jiang Y, Dai M, Lu J, Xu M, Li Y, Hu N, Li J, Mi S, Chen C, Li G, Mu Y, Zhao J, Kong L, Chen J, Lai S, Wang W, Zhao W, Ning G, 2010 China Noncommunicable Disease Surveillance Group (2013). Prevalence and control of diabetes in Chinese adults. JAMA.

[7] Ji L, Hu D, Pan C, Weng J, Huo Y, Ma C, Mu Y, Hao C, Ji Q, Ran X, Su B, Zhuo H, Fox KAA, Weber M, Zhang D, CCMR Advisory Board, CCMR-3B STUDY Investigators (2013). Primacy of the 3B approach to control risk factors for cardiovascular disease in type 2 diabetes patients. Am J Med.

[8] Jia W, Zhang P, Duolikun N, Zhu D, Li H, Bao Y, Li X, Liu Y, ROADMAP study group (2020). Study protocol for the road to hierarchical diabetes management at primary care (ROADMAP) study in China: a cluster randomised controlled trial. BMJ Open.

[9] Workgroup on Hypoglycemia‚ American Diabetes Association (2005). Defining and reporting hypoglycemia in diabetes: a report from the American Diabetes Association Workgroup on Hypoglycemia. Diabetes Care.

[10] EuroQol G (1990). EuroQol--a new facility for the measurement of health-related quality of life. Health Policy.

[11] Liu GG, Wu H, Li M, Gao C, Luo N (2014). Chinese time trade-off values for EQ-5D health states. Value Health.

[12] Toobert DJ, Hampson SE, Glasgow RE (2000). The summary of diabetes self-care activities measure: results from 7 studies and a revised scale. Diabetes Care.

[13] Ji L, Zhang P, Weng J, Lu J, Guo X, Jia W, Yang W, Zou D, Zhou Z, Pan C, Gao Y, Li X, Zhu D, Li Y, Wu Y, Garg SK (2015). Observational Registry of Basal Insulin Treatment (ORBIT) in patients with type 2 diabetes uncontrolled by oral hypoglycemic agents in China--study design and baseline characteristics. Diabetes Technol Ther.

[14] Normand ST, Landrum MB, Guadagnoli E, Ayanian JZ, Ryan TJ, Cleary PD, McNeil BJ (2001). Validating recommendations for coronary angiography following acute myocardial infarction in the elderly: a matched analysis using propensity scores. J Clin Epidemiol.

[15] van Buuren S (2007). Multiple imputation of discrete and continuous data by fully conditional specification. Stat Methods Med Res.

[16] Vansteelandt S, Daniel RM (2014). On regression adjustment for the propensity score. Stat Med.

[17] Benedetto U, Head SJ, Angelini GD, Blackstone EH (2018). Statistical primer: propensity score matching and its alternatives. Eur J Cardiothorac Surg.

